# An objective system for appraising clear aligner treatment difficulty: clear aligner treatment complexity assessment tool (CAT–CAT)

**DOI:** 10.1186/s12903-020-01300-6

**Published:** 2020-11-10

**Authors:** Hu Long, Zhouqiang Wu, Xinyu Yan, Qingxuan Wang, Lu Liu, Yan Wang, Fan Jian, Lina Liao, Xiaolong Li, Wenli Lai

**Affiliations:** Department of Orthodontics, State Key Laboratory of Oral Diseases and National Clinical Research Center for Oral Diseases, West China Hospital of Stomatology, Sichuan University, Chengdu, China

**Keywords:** Clear aligner, Treatment difficulty, Clear aligner treatment complexity assessment tool, CAT–CAT

## Abstract

**Background:**

Recent years have witnessed a remarkable evolution of clear aligner technology and clear aligners are becoming more and more versatile in treating orthodontic patients. The aim of this study was to develop an objective evaluation system for assessing clear aligner treatment difficulty.

**Methods:**

A total of 120 eligible patients (100 patients for developing and testing the evaluation system and 20 patients for validating this system) were recruited in this retrospective cross-sectional study. Based on clinical data (dental models, radiographs and photographs), complexity levels of cases were evaluated by two experts and regarded as the gold standard. Difficulty scores were determined through an evaluation system encompassing three domains (dental model analysis, radiographic examinations and clinical examinations). The reliability of the evaluation system was examined through analyzing the agreement between complexity levels and difficulty scores. Moreover, multivariable linear regression test was used to examine the independent association of each variable (e.g. overbite and crowding) with the complexity level.

**Results:**

The results revealed that the assessment of treatment difficulty by this objective evaluation system substantially matched the gold standard (R^2^ = 0.80). The multivariable regression test revealed that complexity level was significantly associated with difficulty score (p < 0.001), age (p = 0.015), tooth extraction (p < 0.001), treatment stage (p < 0.01) and the number of difficult tooth movement (p = 0.005). This objective evaluation system elaborated in this study was viable and reliable in appraising clear-aligner treatment difficulty in clinical practice.

**Conclusions:**

We suggest orthodontists and general practitioners use this objective evaluation system (CAT-CAT) to appraise clear aligner treatment difficulty and to select appropriate clear aligner patients.

## Background

Recent years have witnessed a dramatic evolution of clear aligner technology and numerous innovations have been built into clear aligner [1]. Compared with traditional brackets and wires, clear aligner appeals to orthodontic patients for its advantages of invisibility, comfort and esthetics [2–6]. This renders more and more practitioners, especially general practitioners, to use clear aligner for orthodontic patients [7]. However, different opinions exist on the ability and versatility of clear aligner in treating different types of malocclusions [8, 9], which is partly attributed to different levels of education and training received by practitioners. It has been reported that practitioners with different levels of orthodontic education differed in aligner expertise and the management of aligner patients [7]. Moreover, practitioners with more advanced orthodontic training are better at recognizing case complexity and eliminating potential risks [10]. Thus, to guarantee successful treatment of clear aligner patients, the expertise of recognizing case complexity of aligner patients is very important.

Although numerous evaluation systems have been designed for the assessment of case complexity for traditional fixed appliances, such as peer assessment rating (PAR) and ABO discrepancy index (DI) [11, 12], none of them is developed for clear aligner. Considering that clear aligner has a distinct biomechanical system and achieves different types of tooth movements with varying degrees of predictability [13–16], an evaluation system of case complexity specifically for clear aligner treatment is urgently needed for both orthodontists and general practitioners.

Therefore, the aim of this study was to develop an evaluation system for the assessment of case complexity for clear aligner patients. We hypothesized that our evaluation system would demonstrate high viability and reliability in clinical practice.

## Methods

### Study design

Our study was a retrospective study. Two groups of patients were included in this study. The first group comprising a total of 100 consecutive patients receiving clear aligner treatments from Jan 2016 to Dec 2016 was recruited from a patient pool (treated by Prof. Lai) from West China Hospital of Stomatology, Sichuan University, Chengdu, China to develop and test the evaluation system. Patient recruitment was not completed until a variety of malocclusion types (deepbite, openbite, crossbite, crowding, skeletal anomaly, etc.) were included. Moreover, the second group of 20 eligible patients (Jan 2017 to Feb 2017) were included in this study to examine the external validity of the evaluation system.

### Subjects

The inclusion criteria were patients receiving Invisalign® (San Jose, California, USA) clear aligner treatment and both genders were included. The exclusion criteria included orofacial syndromes and unwillingness to participate. All the eligible subjects were consecutively included in our study.

### Data collection

Prior to orthodontic treatment, clinical photographs, panoramic radiograph, lateral radiograph and dental impressions were taken for all the subjects. Dental study casts were made from the dental impressions. Furthermore, clear aligner treatment stages and the number of difficult tooth movements were recorded from the clear aligner software (Clincheck, San Jose, USA). Specifically, difficult tooth movement was automatically detected by the clear aligner software system based on predicted tooth movements (e.g., molar protraction > 5 mm), and the total number of teeth with difficult tooth movement was counted for each patient.

### Ethical approval

This study was approved by the Ethical Committee of West China Hospital of Stomatology, Sichuan University (WCHSIRB-OT-2019-086). Informed consent was obtained from all the patients above 18 yrs old, or from the patients and their parents for those under 18 yrs old.

### Evaluation of complexity level by two experts

Based on the clinical data (clinical photographs, radiographs and dental casts), complexity levels of patients receiving clear aligner treatment were assessed by two experts (WL & HL) independently and in duplicate. The complexity levels assessed by the two experts ranged from one to ten, with one being the easiest and ten being the most difficult. The results of complexity levels assessed by the experts were regarded as the gold standard. Specifically, complexity level was comprehensively determined according to pre-treatment data (crowding, overjet, overbite, etc.) and the amount of predicted tooth movement (incisor retraction, molar mesialization, molar distalization, premolar derotation, etc.).

### Difficulty scoring through the evaluation system

The difficulty scoring was conducted by two independent evaluators (ZW & QW). The complexity assessment tool was an objective evaluation system that encompassed three major domains: dental model analysis, radiographic examinations and clinical examinations. The assessment tool is detailed in Table 1. In brief, the domain of dental model analysis mainly included overjet, overbite, crowding, molar relationship, posterior teeth and spacing. The domain of radiographic examinations mainly encompasses ANB angle, U1-SN angle and SN-MP angle. ANB is a radiographic index for assessing relative sagittal positions between upper and lower jaws, SN-MP is an index for evaluating mandibular plane angle, and U1-SN denotes upper incisor proclination. Furthermore, the domain of clinical examinations mainly included E-line and gummy smile. Other clinical examinations were also taken into consideration, including chin deviation (if surgery), occlusal canting, periodontitis, temporomandibular disease (TMD) and generalized caries. For each domain, different weights were added onto different items.

**Table 1:**
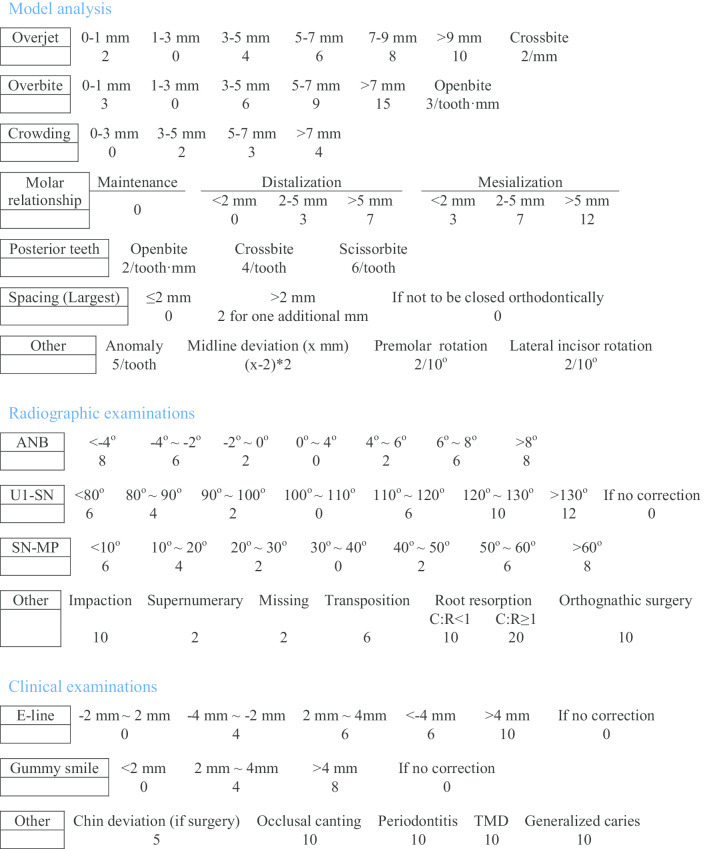
Clear aligner treatment complexity assessment tool (CAT–CAT)

For each subject, difficulty scoring was performed through the complexity assessment tool, and the sum of the scores of all the items was regarded as the final difficulty score.

### Reliability of the evaluation system

The reliability of the complexity assessment tool was determined through analyzing the agreement between the gold standard (complexity levels evaluated by the expert) and the difficulty scores obtained through the assessment tool.

### Statistical analysis

Intra-class coefficient (ICC) was used to test the agreement of complexity levels between the two experts and the agreement of difficulty scores between the two evaluators. The reliability of the assessment tool was analyzed through Pearson’s correlation test. Moreover, multivariable linear regression test was used to examine the independent association of each variable (e.g., overbite and crowding) with the complexity level. All the statistical analyses were performed in GraphPad Prism 7.0 and SPSS 21.0. A p value less than 0.05 was considered statistically significant.

## Results

### Patients

For the first group (n = 100), their ages ranged from 10 to 48, with a female predominance (86%). Among them, 64% were non-extraction patients (n = 64) and the remaining 36% were extraction patients (n = 36). Aligner stages ranged from 12 to 98, with a mean of 39. Moreover, the number of difficult tooth movement ranged from 0 to 20, with a mean of 7.3. Similar results were found for the second group (n = 20).

### Evaluation of complexity levels

**Fig. 1:**
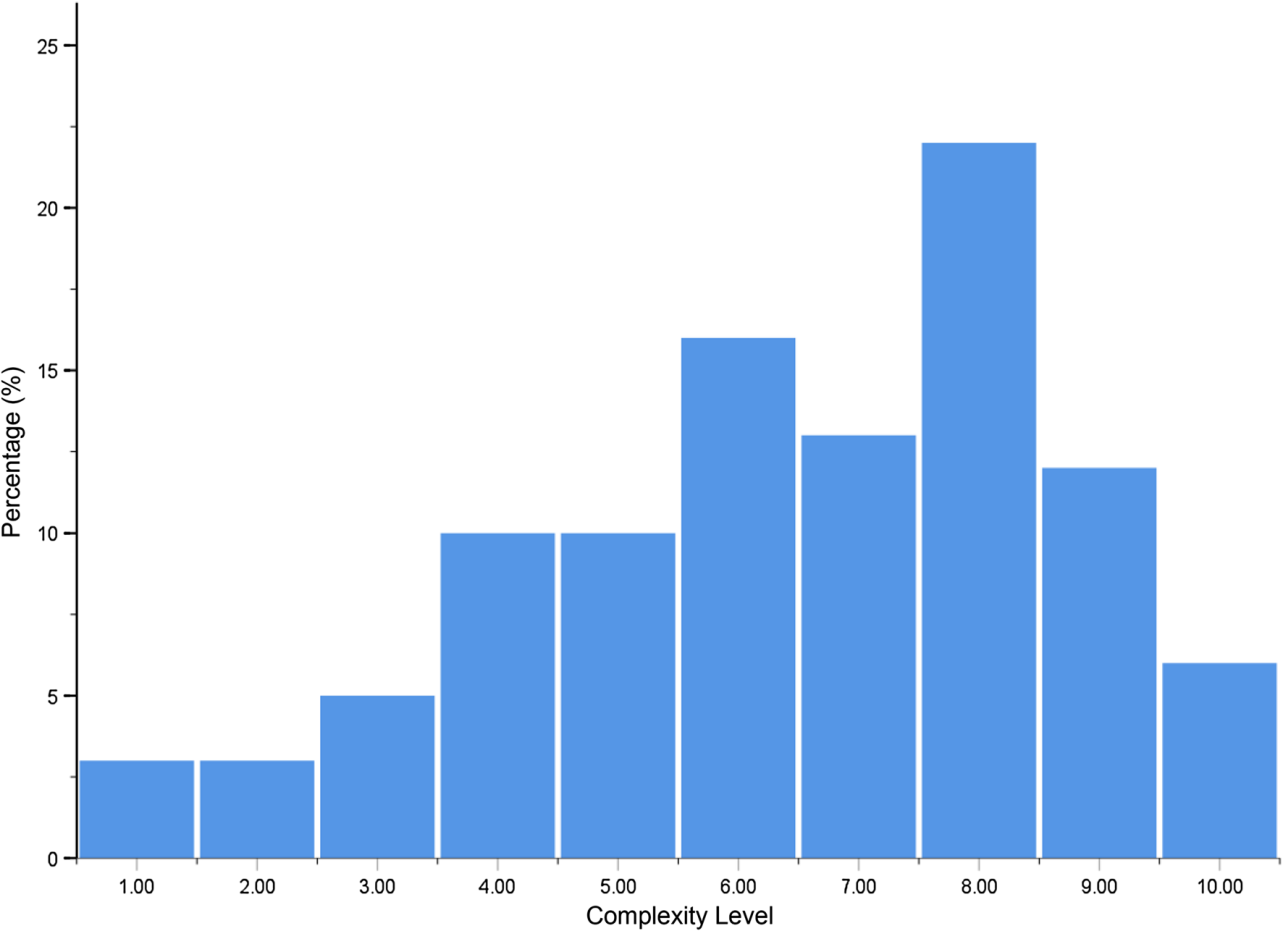
The percentage distribution of complexity levels as assessed by orthodontic experts

The complexity levels assessed by the two experts were 6.5 ± 2.3. The ICC was 0.92, indicating that the evaluation of treatment complexity by the two experts were in substantial agreement. The percentage of different complexity levels is displayed in Fig. 1.

### Evaluation of difficulty scores

**Fig. 2:**
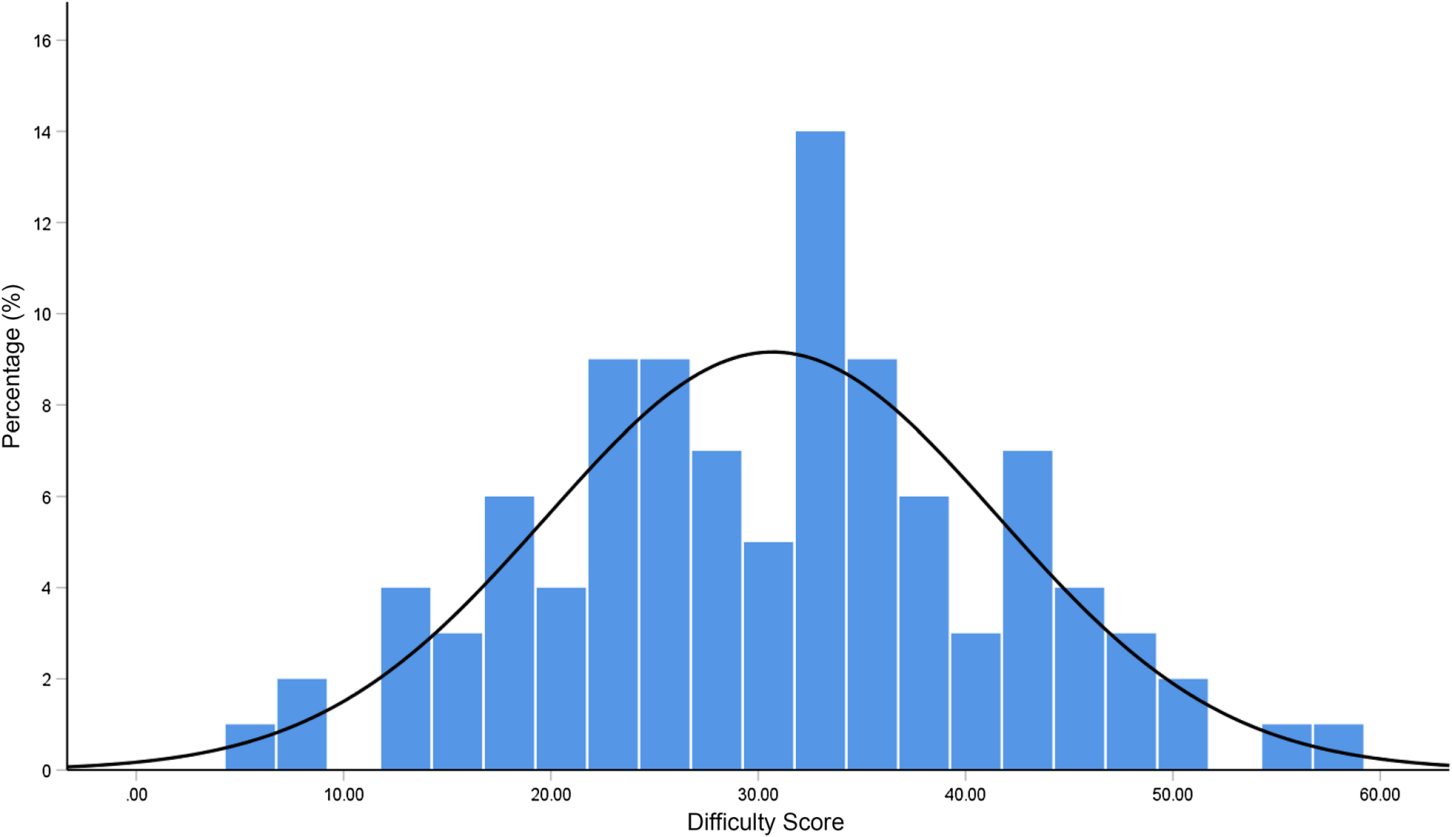
The percentage distribution of difficulty scores as evaluated through clear aligner treatment complexity assessment tool (CAT–CAT)

The difficulty scores ranged from 6 to 59, with a mean of 30.7 ± 10.9. The reliability test showed that the results assessed by the two evaluators were in substantial agreement (ICC = 0.86). The percentage of different difficulty scores is depicted in Fig. 2. Shapiro–Wilk normality test revealed that the difficulty scores of the subjects were normally distributed (p = 0.99).

### Agreement between complexity level and difficulty score

**Fig. 3:**
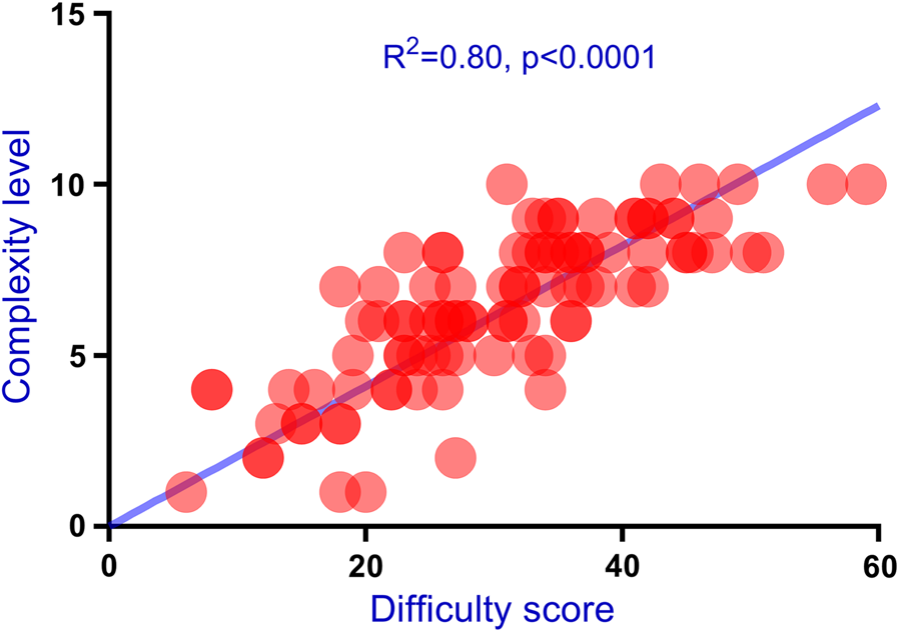
Agreement between complexity level and difficulty score. Pearson’s correlation test revealed that the two items were significantly correlated (R^2^ = 0.80)

As depicted in Fig. 3, Pearson’s correlation test revealed that complexity level and difficulty score were significantly correlated (R^2^ = 0.80, p < 0.001). Moreover, likewise, for the second group, complexity level was significantly correlated with difficulty score (R^2^ = 0.82, p < 0.001).

### Multivariable regression test

The results revealed that complexity level was significantly associated with difficulty score (β = 0.13, 95% CI 0.11 ~ 0.16; p < 0.001), age (β = −0.04, 95% CI −0.07 ~ −0.01; p = 0.015 < 0.01), tooth extraction (β = 1.14, 95% CI 0.61 ~ 1.67; p < 0.001), treatment stage (β = 0.025, 95% CI 0.007 ~ −0.043; p = 0.009 < 0.01) and the number of difficult tooth movement (β = 0.09, 95% CI 0.03 ~ 0.15; p = 0.005 < 0.05). However, it was not associated with gender (p = 0.22 > 0.05).

We further performed multivariable linear regression test to analyze the association between the complexity level and the items in the evaluation system. As displayed in Table 2, we found that complexity level was significantly correlated with overjet (β = 0.18, 95% CI 0.09 ~ 0.27; p < 0.001), overbite (β = 0.14, 95% CI 0.08 ~ 0.20; p < 0.001), crowding (β = 0.43, 95% CI 0.24 ~ 0.61; p < 0.001), molar relationship (β = 0.23, 95% CI 0.14 ~ 0.33; p < 0.001), posterior teeth (β = 0.17, 95% CI 0.11 ~ 0.24; p < 0.001), other model analysis (β = 0.15, 95% CI 0.08 ~ 0.22; p < 0.001), ANB (β = 0.13, 95% CI 0.04 ~ 0.22; p = 0.006 < 0.05), U1-SN (β = 0.23, 95% CI 0.14 ~ 0.32; p < 0.001), SN-MP (β = 0.24, 95% CI 0.03 ~ 0.44; p = 0.02 < 0.05), E-line (β = 0.21, 95% CI 0.14 ~ 0.29; p < 0.001) and other clinical examinations (β = 0.08, 95% CI 0.01 ~ 0.16; p = 0.03 < 0.05). Yet, it was not associated with spacing (p = 0.96 > 0.05), other radiographic analysis (p = 0.23 > 0.05) or gummy smile (p = 0.18 > 0.05).

**Table 2:** Multivariable regression analysis of the association between multiple indices and complexity level

Index	Regression coefficient (β) (95% CI)	p value
*Dental model analysis*		
Overjet*	0.18 (0.09, 0.27)	< 0.001
Overbite*	0.14 (0.08,0.20)	< 0.001
Crowding*	0.43 (0.24, 0.61)	< 0.001
Molar relationship*	0.23 (0.14, 0.33)	< 0.001
Spacing (the largest)	−0.01 (−0.17, 0.16)	0.96
Posterior teeth*	0.17 (0.11, 0.24)	< 0.001
Other model analysis*	0.15 (0.08, 0.22)	< 0.001
*Radiographic examinations*		
ANB*	0.23 (0.04, 0.22)	0.006
U1-SN*	0.23 (0.14, 0.32)	< 0.001
SN-MP*	0.24 (0.03, 0.44)	0.02
Other radiographic examinations	0.06 (−0.04, 0.17)	0.23
*Clinical examinations*		
E-line*	0.21 (0.14, 0.32)	< 0.001
Gummy smile	0.18 (−0.09, 0.45)	0.18
Other clinical examinations*	0.08 (0.01, 0.16)	0.03

*p < 0.05 indicates statistical significance

## Discussion

Clear aligner differs from conventional fixed appliances in biomechanics. For clear aligners, tooth movements are achieved through compressive force on teeth produced by elastic changes of aligners. In contrast, teeth are moved through both compressive and traction forces generated by the interaction between brackets and archwires. Moreover, distinct from fixed appliances, the clear aligner system suffers from a significant disadvantage: teeth may “escape” from the aligners (off-tracking) and force applications cannot be adequately applied on these teeth [17]. This phenomenon results in varying degrees of predictability for different types of tooth movements, with molar distalization being the most predictable (88%) while incisor extrusion being the least (30%) [13–16]. The evaluation system of treatment complexity elaborated in this study was designed specifically for the clear aligners and our results revealed that the assessment of treatment complexity by this objective evaluation system substantially matched the gold standard results by the two experts (R^2^ = 0.80).

To date, several evaluation systems for assessing treatment complexity are available for conventional fixed appliances, e.g., PAR and DI [11, 12]. PAR system appraises treatment complexity based on model analysis only, and DI system evaluates treatment complexity through analysing dental models and radiographs. However, neither of above systems includes soft tissue analysis for the assessment of treatment complexity. The evaluation system described in this study took all the three tissues (dental, skeletal and soft tissues) into consideration. The scoring rules in this evaluation system for clear aligners were based on those in PAR and DI with modifications according to the unique characteristics of clear aligner treatment. Specifically, less weight was assigned to the tooth movement that was easy for clear aligners (e.g., molar distalization), while more weight to difficult tooth movement with clear aligners (e.g., molar mesialization). On the other hand, all the evaluation systems, including our present one, assess treatment difficulty through summing up difficulty points of all independent items, (e.g., overbite, overjet and molar relationship), but PAR and DI systems fail to evaluate treatment difficulty in a dynamic way. For example, a full-cusp Class II molar relationship is considered to be more difficult than Class I relationship. In effect, in clinical scenarios, full-cusp Class II molar relationship is clinically acceptable and molar movement is not necessarily required. Thus, for molar movement, the treatment difficulty is the same for a Class I relationship and a full Class II relationship. The only difference was the overjet: a patient with full-cusp Class II were more difficult due to a larger overjet that should be corrected rather than due to molar relationship. Thus, no point was added for patients whose molar relationship will be maintained in our present evaluation system. Therefore, molar relationship was evaluated in a dynamic way in our evaluation system, rather than a simple classification of molar relationship.

The multivariable regression test revealed that complexity level was positively correlated with difficulty score (β = 0.13, 95% CI 0.11 ~ 0.16; p < 0.001), indicating that complexity level will be increased by 0.13 if difficulty score is increased by one. Moreover, we found that complexity level was positively associated with tooth extraction and the number of difficult tooth movement. Although clear aligners are able to manage extraction patients with good treatment outcomes [18, 19], premolar extractions followed by anterior teeth retraction requires meticulous design of aligner biomechanics, which will definitely increase treatment complexity. Difficult tooth movement was defined by the clear aligner software based on predicted distances of movement for each tooth, e.g., molar intrusion greater than 5 mm. Conceivably, a higher complexity level is anticipated with a larger number of difficult tooth movements. Interestingly, we found that complexity level was negatively associated with patient age (β = −0.04, p = 0.015 < 0.05). This may be attributed to a selection bias that adult patients with high treatment complexity were not included in this study given that patients with greater age had smaller number of difficult tooth movement (p = 0.01 < 0.05).

For the domain of model analysis, the multivariable regression test revealed that complexity level was positively associated with all items except for spacing (Table 2). A large overjet requires premolar extractions and subsequent upper anterior retraction, while large overbite requires large amounts of lower incisor intrusions. All of these tooth movement types are considered to be difficult for clear aligners. Therefore, we put more weights on these two items (e.g., 10 points will be added for a patient with an overbite greater than 9 mm and 9 points for a patient with an overbite of 6 mm). Treatment complexity is higher among patients with more crowding (p < 0.001). However, we did not put much weight on this item since severe crowding could be easily resolved through premolar extraction and subsequent minimal incisor retraction (most of the extraction space is used for resolving crowding rather than incisor retraction). As mentioned above, we evaluated molar relationship in a dynamic way: zero point was added for patients with molar relationship maintenance (Class I, full-cusp Class II or full-cusp Class III). Considering molar mesialization is more difficult than molar distalization with clear aligners [20], we put more weights on molar mesialization. For posterior teeth, all the three types of malocclusions (openbite, crossbite and scissorbite) are difficult to treat and thus we added much weight on this item, e.g., 10 points will be added for patients with a posterior tooth with a 5-mm openbite (2 pts/tooth·mm). For the item of spacing, we only analyzed the largest space since patients with one space of 5 mm will be more difficult to treat than those with several small spaces totaling 5 mm (if the spaces are to be closed orthodontically). Moreover, if a space will not be closed orthodontically (e.g., closure through implants), no point will be added. For other model analyses, tooth anomaly, midline deviation, premolar rotation and incisor rotation were also correlated with complexity level. As is well documented, tooth movement is achieved through elastic changes of aligners, and adequate aligner wrapping is critical for achieving the predicted tooth movement. Any tooth anomaly will reduce the adequacy of aligner wrapping, making tooth movement less predictable. Hence, five points will be added for each abnormal tooth. It has been reported that a midline deviation less than 2 mm was accepted by the general population [21]. Thus, only the amount of midline deviation greater than 2 mm was counted in our evaluation system. Rotations of premolar and lateral incisors are difficult to correct in clear aligner system, since premolars are round or oval in shape and lateral incisors are short from the occlusal view (clear aligners are not able to exert adequate tangent forces that are crucial for derotation).

The domain of radiographic examinations encompasses ANB, U1-SN, SN-MP and other radiographic examinations. The deviations of ANB and SN-MP from their normal ranges are indicative of the abnormal development of upper and lower jaws, which will increase clear aligner treatment complexity. Proclined upper incisors require space gaining and subsequent retraction of incisors. Thus, treatment complexity is increased among patients with abnormal U1-SN values. Moreover, tooth impaction, supernumerary teeth, missing teeth, tooth transposition, root resorption and severe skeletal problems needing orthognathic surgery increased clear-aligner treatment difficulty and we put different weights on these items according to their influence on treatment complexity. The multivariable regression test showed that complexity level was associated with all items in the domain of radiographic examinations except for other radiographic examinations. This could be attributed to the fact that small number of patients had these other radiographic problems (e.g., impaction), making this index (other radiographic examinations) similar among patients with different complexity levels. Thus, further studies with larger sample sizes are warranted.

The domain of clinical examinations had three indices: E-line, gummy smile and other clinical examinations. The multivariable regression test found that complexity level was correlated with E-line and other radiographic examinations, while not with gummy smile. Likewise, this may be due to that small number of patients required gummy smile correction. An abnormal E-line (e.g., 4 mm denotes lips are protrusive by 4 mm in reference to E-line) requires space gaining and anterior teeth retraction, thereby increasing aligner case difficulty. Moreover, all of the indices (chin deviation, occlusal canting, periodontitis, TMD and generalized caries) in “other clinical examinations” increased clear aligner treatment complexity. Thus, we put different weights on these indices.

The patients in the second group were included for examining the external validity of the complexity assessment tool. Our results revealed that the difficulty scores (obtained through assessment tool) were significantly correlated with complexity level (assessed by experts) with an R^2^ of 0.82. This finding was consistent with that from the first group, indicative of the external validity of this assessment tool. However, the limitation of this pilot study was relatively small sample size. Thus, future studies with larger sample sizes in different clinical settings among different races are warranted to further confirm the validity of this complexity assessment tool.

## Conclusion

The objective evaluation system (CAT-CAT) elaborated in this study is viable and reliable in appraising clear aligner treatment difficulty in clinical practice. We suggest orthodontists and general practitioners use this evaluation system to appraise clear aligner treatment difficulty and to select appropriate clear aligner patients.

## Data Availability

The datasets used and/or analyzed during the current study are available from the corresponding author on reasonable request.

## Abbreviations

ICC: Intra-class correlation coefficient
PAR: Peer assessment rating
DI: Discrepancy index
CAT–CAT: Clear aligner treatment complexity assessment tool
